# Coding-complete genome sequence of a lumpy skin disease virus (LSDV) isolate from Rajshahi, Bangladesh

**DOI:** 10.1128/mra.01391-25

**Published:** 2026-03-12

**Authors:** Md. Mukter Hossain, Ho-Seong Cho, Syed Muktadir Al Sium, Ruhena Begum, Md Bashir Uddin

**Affiliations:** 1 Department of Medicine, Sylhet Agricultural University 399806, Sylhet, Bangladesh; 2 College of Veterinary Medicine and Bio-safety Research Institute, Jeonbuk National University 26714, Iksan, Republic of Korea; 3 Bangladesh Council of Scientific and Industrial Research 130051, Dhaka, Bangladesh; Portland State University, Portland, Oregon, USA

**Keywords:** LSDV, whole genome, cattle, Bangladesh

## Abstract

We report a coding-complete genome sequence of a lumpy skin disease virus (LSDV) isolate (SAUVM-78) collected on 11 October 2021, during a clinical outbreak in Rajshahi, Bangladesh. This resource supports ongoing genomic surveillance of Capripoxviruses in South Asia.

## ANNOUNCEMENT

Lumpy skin disease virus (LSDV; genus *Capripoxvirus*, family *Poxviridae*) is an emerging transboundary pathogen of cattle with substantial economic impact across Asia and other regions. Despite recent outbreaks, genomic resources from South Asia remain comparatively limited (1–3). As of 19 November 2025, GenBank lists only 10 coding complete LSDV genomes from Bangladesh. Here, we present a coding-complete genome of LSDV isolate SAUVM-78 from Bangladesh to support surveillance and comparative genomics.

Pus from skin nodules was aseptically collected from Bos indicus cattle exhibiting characteristic LSD lesions in Rajshahi, Bangladesh, on 11 October 2021 (locality: 24.368000° N, 88.599500° E). Specimens were placed in viral transport medium containing antibiotics (penicillin and streptomycin) to inhibit bacterial contamination. Samples were immediately transported on ice to the Laboratory of Medicine, Sylhet Agricultural University, and stored at −80°C until further processing. No cell-culture amplification was performed prior to sequencing. Viral nucleic acids were extracted using the AddPrep Viral Nucleic Acid Extraction Kit (ADDBIO, Republic of Korea) from direct clinical sample, that is, pus from skin nodules, following the manufacturer’s protocol. Sequencing libraries were prepared with the NEBNext Ultra II DNA Library Prep Kit (New England Biolabs, China). Libraries were sequenced on an Illumina Novaseq 6000 with paired-end 2 × 150 bp reads.

All raw reads (*n* = 17,383,080) were subjected to quality control prior to reference-guided assembly. Quality assessment using FastQC v0.11.9 and MultiQC v1.13 revealed that >90% of bases had Phred scores >Q30 (4, 5). Low-quality reads and adapter sequences were trimmed using the quality trimming function implemented in CLC Genomics Workbench v8.0 with default parameters against LSDV/02/KASH/IND/2022 (OQ588787.1) (6) with a similarity fraction of 0.9**,** mismatch/indel costs of 2/6, and local realignment enabled. Consensus sequences were generated using the “Extract consensus sequence” function with a low-coverage threshold of 1, removal and joining of low-coverage regions**,** conflict resolution by vote with quality scores, and a minimum coverage of 30×. Reads below the quality threshold after trimming were discarded. The remaining high-quality reads were then used for reference-guided assembly. The final assembly yielded a coding-complete genome of 151,036 bp with an average coverage of 90.06×.

**Fig 1:**
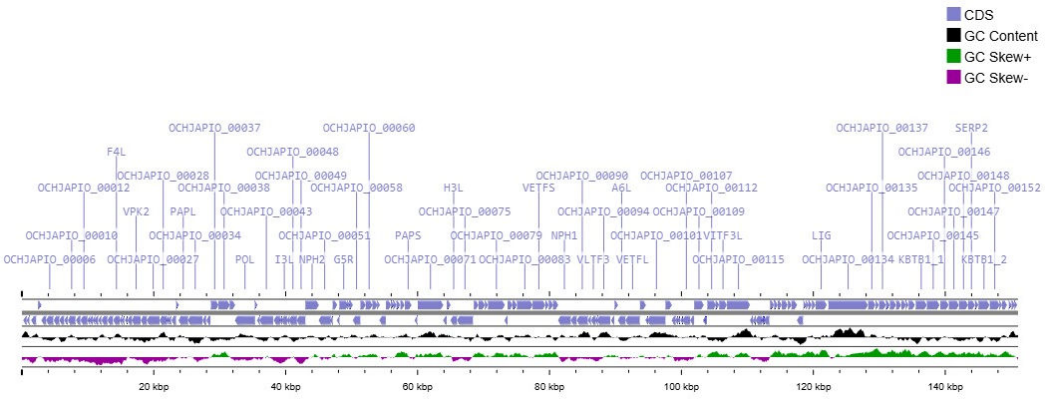
Genome map of the lumpy skin disease virus (LSDV) isolate SAUVM-78 generated using Proksee. The map displays the coding-complete genome organization (151,036 bp), including predicted 157 coding sequences (CDSs) annotated by Prokka v1.14.6, GC content, and GC skew across the genome. CDSs are shown according to their genomic orientation, while GC content (25.9%) and GC skew are represented in the inner rings.

Genome annotation predicted 157 coding sequences (CDSs) using Prokka v1.14.6, supported by InterProScan v5.59 and validated with BLASTp against the UniProtKB and NCBI nonredundant databases (7–9). A linear genome map was generated with Proksee (Fig. 1) (10). Lineage and phylogenetic placement were performed using BLASTn and phylogenetic analyses of conserved LSDV markers (P32, GPCR, RPO30, and Ankyrin repeat gene), using MEGA11 with maximum likelihood methods (11). All software used default parameters unless otherwise specified.

Comparison with the reference genome LSDV NI-2490 (GenBank accession no. NC_003027.1) revealed 10 nucleotide variations in the SAUVM-78 genome, including six single nucleotide substitutions (20C>A, 28653G>A, 75262C>A, 88961G>T, 133173T>A, and 150754G>T), two deletions (13110delT and 116105delA), and two insertions (23369_23370insT and 114039_114040insA). BLASTn analysis identified LSDV isolate OP688129.1, also collected in Bangladesh (from Mymensingh) in 2021, as the closest genomic match. All 10 mutations found in SAUVM-78 were shared with OP688129.1, suggesting a close evolutionary relationship and possible circulation of a common strain in the region. However, OP688129.1 harbored three additional mutations (12080_12081insT, 45482C>T, and 146714_146715insA) that were not detected in SAUVM-78, indicating isolate-specific differences and highlighting the ongoing microevolution of LSDV within Bangladesh.

## Data Availability

The coding-complete genome sequence of LSDV isolate SAUVM-78 has been deposited in GenBank under accession number PP979138.1, and the raw reads are available under SRA accession SRR35783399 within BioProject number PRJNA1345064 (https://trace.ncbi.nlm.nih.gov/Traces/?run=SRR35783399).
